# Upregulation of heme oxygenase-1 expression may facilitate memory and learning in mice

**DOI:** 10.3892/etm.2013.995

**Published:** 2013-03-08

**Authors:** YU CHEN, FANG HUANG, DAYONG WANG, ZHENG WENG, ZHONGLIANG DENG

**Affiliations:** 1 Department of Orthopedics, The Ninth People’s Hospital of Chongqing, Chongqing 400700;; 2 Department of Respiratory Medicine, The Third People’s Hospital of Chongqing, Chongqing 400014;; 3 Department of Orthopedics, The Second Affiliated Hospital of Chongqing Medical University, Chongqing 400016, P.R. China

**Keywords:** heme oxygenase-1, learning, memory, upregulation

## Abstract

Heme oxygenase (HO)-1 is highly expressed in the hippocampus. Its expression is induced by many factors including hemes, whose metabolites play an important role in neuron protection and learning development. In the present study, the correlation between HO-1 and learning ability was investigated in mice. Behavioral tests were used to evaluate the effects of altering HO-1 on learning ability in mature mice. In order to determine the function of HO-1 in the immature mice, a dark-reared model was constructed. Either the HO-1 inducer hemin or the HO-1 inhibitor Zn protoporphyrin IX (ZnPPIX) was injected into the left lateral ventricle prior to a behavior test. Results showed that neither hemin nor ZnPPIX affected the learning ability of adult mice reared in normal conditions. The hippocampal HO-1 of dark-reared mice was decreased while it was increased in the behavioral training group. In general, HO-1 had no effect on established learning ability but it may be upregulated by behavioral training and is beneficial for the development of memory and learning ability in neonatal mice.

## Introduction

It is well known that heme oxygenase (HO) functions as the rate-limiting enzyme in heme degradation, which takes place in the endoplasmic reticulum. There are three isoforms of HO in the body, namely HO-1, 2 and 3 (1). HO has been reported to be present in all tissues and is located in microsomes (2). Recently HO-1 and 2 have been shown to be present in mitochondria (3,4). HO-1 is inducible by inflammatory cytokines and oxidants, including nitric oxide (NO), whereas HO-2 and 3 are expressed constitutively (1,5). It was reported that HO-1 mRNA was present in different regions of the brain, especially the hippocampus and cerebellum (6). Certain studies have reported impaired spatial navigation learning ability in transgenic mice overexpressing HO-1 (7). Other studies have shown an age-related decrease in HO-1 expression to be present in specific brain regions, including the hippocampus. Further, neotrofin (AIT), a cognitive-enhancing and neuroprotective drug, was found to cause a robust increase in HO-1 immunoreactive protein in the same regions (8). Alzheimer’s disease (AD) is a common age-associated dementia featuring progressive loss of neurons and synapses, gliosis and the accumulation of intra- and extracellular protein deposits. In individuals with AD, the increasing impairment of learning and memory eventually leads to a definitive diagnosis. It appears that oxidative injury is central in the pathogenesis, even prior to the appearance of amyloid deposits (9,10). Intrahippocampal injection of a lentiviral vector expressing nuclear factor (erythroid-derived 2)-like 2 (Nrf2) was found to improve spatial learning in a mouse model of Alzheimer’s disease and Nrf2 gene transfer was associated with a robust reduction in astrocytic but not microglial activation as well as the induction of the Nrf2 target gene HO-1 (11). It appears that the role of HO-1 in the modulation of learning ability is complex. The aim of the present study was to elucidate the correlation between HO-1 and learning.

## Materials and methods

### Subjects

Thirty-five nine-day-old male NIH mice were housed in polyethylene cages (five mice per cage) and fed with standard chow pellets and drinking water until they reached 55 days old. This study was carried out in strict accordance with the recommendations in the Guide for the Care and Use of Laboratory Animals of the National Institutes of Health. The animal use protocol was reviewed and approved by the Institutional Animal Care and Use Committee (IACUC) of the Second Affiliated Hospital of Chongqing Medical University, China. All experiments were conducted in accordance with the guidelines of Chongqing Medical University and the Animal Care Committee.

### Surgery

Following anesthesia with 4% chloral hydrate, a stainless steel cylindrical cannula (outer diameter, 0.6 mm; inner diameter, 0.4 mm) with a stopper was implanted so that the tip of the cannula was in the left lateral ventricle (1.3 mm lateral to the midline, 0.3 mm posterior to the bregma, 2.0 mm ventral to the dura). The cylindrical cannula was fixed with dental cement mixed with fast condensation glue. During the surgery, body temperature was monitored and maintained at 37±0.5°C.

### Behavioral test/training

In the step-down test, mice were placed on the platform. If the mice stepped down onto the floor they received a 36 V AC foot shock. Mice typically jumped quickly onto the platform to avoid the electric stimulation. The error number (more than two extremities touching the grid) and the electric shock time were recorded for 10 min. A day later, the mice were placed on the platform again but without electrifying the grid. The step-down latency and the time remaining on the platform were recorded over a 5 min period.

In the step-through test, mice were first placed in the illuminated compartment. Mice would typically enter the dark compartment and encounter a 42 V AC foot shock. The time taken to enter the dark compartment from the illuminated one was recorded. Memory retention trials were performed 24 h later by placing the mice into the illuminated compartment and measuring the dark component entry latency.

In the Morris water maze test, the mice were placed in the maze for 60 sec without a platform. A hidden platform was then placed in the center of one quadrant. The mice were given four trials per day for four consecutive days. In each trial, the mice were placed in the water, facing the edge of the pool in one of the quadrants. The start position varied in a quasi-random fashion, so that the mice never started at the same place in two consecutive trials. The mice were then allowed to seek the hidden platform. If a mouse failed to find the platform in 60 sec, it was placed on the platform for another 15 sec. There was an inter-trial interval of 30 min. On the fifth day, the mice were placed in a randomly selected quadrant and the time to reach the hidden platform and swim speed were recorded.

### Measurement of heme oxygenase activity

Hippocampi were dissected on ice and samples were stored in liquid nitrogen until analysis. The samples were homogenized for 30 sec in three volumes of 0.01 mol/l trisaminomethane hydrochloride (Tris-HCl) containing 0.01 mol/l sucrose and 0.0001 mol/l ethylenediaminetetra-acetic acid disodium salt (EDTANa_2_; 4°C, pH 7.4) using a UP-50H ultra-sonic homogenizer and centrifuged at 4°C at 18,800 ± g for 15 min. Supernatant (0.2 ml) was added to the reaction mixture [2 mmol/l glucose-6-phosphate (Sigma, St. Louis, MO, USA), 0.2 units glucose-6-phosphate dehydrogenase (Sigma), 20 *μ*mol/l hemin, 2 mg rat liver cytoplasm and 0.8 mmol/l nicotinamide adenine dinucleotide phosphate-oxidase (NADPH; Sigma)]. The total volume was 1.0 ml. The mixture was aerobically incubated for 30 min at 37°C in dark conditions and stopped with the addition of 1.0 ml ice-cold chloroform. The production of bilirubin was measured with a double-beam spectrophotometer at ΔOD at 530 nm (extinction coefficient, 40 mM cm^−1^ for bilirubin). Protein concentration was determined using the Coomassie blue method.

### Western blot analysis

Hippocampal tissue was lysed in a solubilization buffer containing 10 mM Tris-HCl (pH 7.4), 0.15 mM NaCl, 1% Nonidet P-40 (Sigma), 0.1% sodium dodecyl sulfate (SDS), 0.001 mg/ml leupeptin (Sigma), 0.001 mg/ml pepstatin (Sigma), 0.001 mg/ml aprotonin (Sigma) and 10% phenylmethylsulfonyl fluoride (PMSF; Sigma) and centrifuged at 4°C at 20,000 × g for 15 min. Protein concentration was estimated using the Bradford method. Protein (40 *μ*g) was subjected to 12% SDS polyacrylamide gel electrophoresis (SDS-PAGE) and transferred to a nitrocellulose membrane. The membrane was blocked for 1 h in phosphate-buffered saline (PBS) containing 5% fat-free milk and 0.2% Tween-20. The blot was incubated for 2 h at 37°C separately with the primary antibody for HO-1 (Sigma), HO -2 (Sigma) and β-actin (Santa Cruz Biotechnology Inc., Santa Cruz, CA, USA), at a 1:400 concentration, followed by incubation for 1 h at 37°C with the secondary antibody (peroxidase-conjugated goat anti-rabbit IgG (Santa Cruz). Immunoreactive bands of HO-1 and 2 were visualized with chemiluminescence reagents. The chemiluminescent signal of the band was detected using a lumino image analyzer (Bio-Rad, Hercules, CA, USA).

### Real-time PCR

Total RNA of hippocampus was isolated according to the RNA isolation kit (Takara Bio, Inc., Shiga, Japan) instructions. Complementary DNA (cDNA) was synthesized according to the instructions of the reverse transcription kit (Toyobo, Osaka, Japan). Amplified DNA was visualized using an ethidium bromide stain with a 2% agarose gel. Results were quantified using the Quantity One analysis software (Bio-Rad). Since glyceraldehyde-3-phosphate dehydrogenase (G3PDH) was the housekeeping gene, amplification signals for HO-1 and 2 mRNA were normalized with the amplification signals of G3PDH mRNA. The HO-1 5′ primer was 5′-AGCACTATGTAAAGCGTCTC-3′ and the 3′ primer was 5′-CGGTCTTAGCCTCTTCTGT-3′ (282 bp). The HO-2 5′ primer was 5′-GACCCAATTCTACCTGTTT-3′ and the 3′ primer was 5′-CCATCCTCCAGGGTTTCT-3′ (207 bp). The G3PDH 5′ primer was 5′-ACCACAGTCCATGCCATCAC-3′ and the 3′ primer was 5′-TCCACCACCCTGTTGCTGTA-3′ (450 bp).

### Study design

All experiments were performed when the mice were fifty days old. Mice were divided into six groups [Hemin, ZnPPIX, artificial cerebrospinal fluid (ACSF), behavior training, dark-reared and control groups]. Each group had five mice, apart from the control group which had ten. All groups were kept in a long daylight cycle condition (16 h light/8 h dark), apart from the dark-reared group which were kept in dark conditions. The hemin, ZnPPIX, ACSF, behavior training and control group (only five mice) underwent the behavior test/training once the intracerebroventricular (icv) injection was completed. Biochemical experiments were conducted after the behavior test.

### Statistical analysis

Data were expressed as means ± standard deviation and were assessed using ANOVA and t-test analyses. P<0.05 was considered to indicate a statistically significant result.

## Results

### Effect of icv injection with various drugs on behavioral test performance

**Figure 1:**
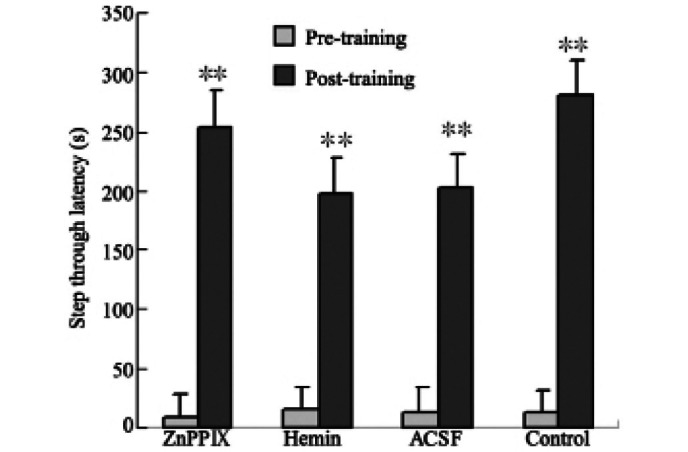
Effect of intracerebroventricular (icv) injection with various treatments on step-through latency (n=10). Training enhanced the step-through latency significantly, but no significant difference in latency was observed among the various pretreatment groups. Student’s t-test, ^**^P<0.01 for post-training vs. pre-training. ZnPPIX, Zn protoporphyrin IX; ACSF, artificial cerebrospinal fluid.

**Figure 2:**
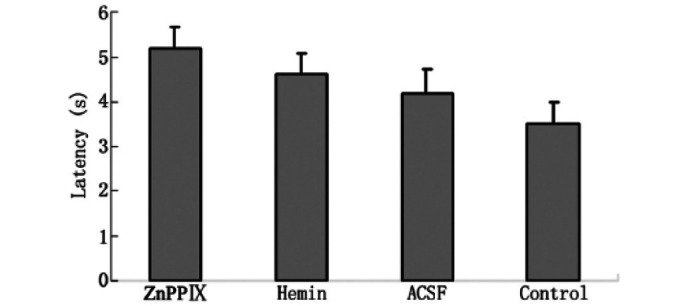
Effect of intracerebroventricular (icv) injection with various treatments on latency for mice to reach the platform in the Morris water maze test (n=10). Various treatments had no effect on the latency for mice to reach the platform. ZnPPIX, Zn protoporphyrin IX; ACSF, artificial cerebrospinal fluid.

As shown in Table I, there was no significant difference among the various groups in the step-down test. In the step-through test, there was a significant difference in the step-through latency between pre-and post-training in each group. No significant difference was observed between the hemin, ACSF, ZnPPIX and control groups (Fig. 1). The data from the Morris water maze test did not indicate any significant differences between the pretreatment groups in swim speed or in the mean latency to reach the platform (Fig. 2, Table II).

### Effect of icv injection with various drugs on HO protein expression

**Figure 3:**
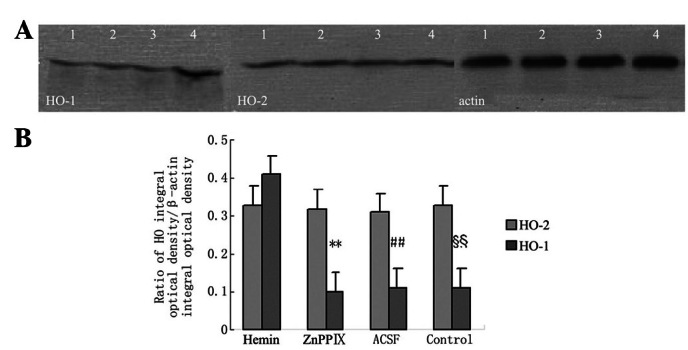
Effect of intracerebroventricular (icv) injection of various treatments on expression of heme oxygenase-1 (HO-1) and 2 (n=5). (A) Expression of HO-1 and 2 detected by western blot analysis. Lane 1, control. Lane 2, Zn protoporphyrin IX (ZnPPIX) icv. Lane 3, artificial cerebrospinal fluid (ACSF) icv. Lane 4, hemin icv. (B) Hemin significantly enhanced the expression of HO-1. Student’s t-test, ^**^P<0.01 for ZnPPIX group vs. hemin group, ^##^P<0.01 for ACSF vs. hemin group; ^§§^P<0.01 for control vs. hemin group.

Fig. 3 shows that hemin induced the expression of HO-1 protein. Hemin and ZnPPIX had no effect on the expression of HO-1. None of the pretreatments affected the expression of HO-2.

### Effect of icv injection with various drugs on HO activity

**Figure 4:**
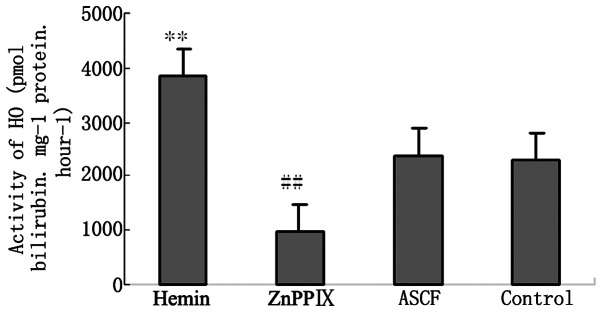
Effect of various treatments injected intracerebroventricularly (icv) on activity of heme oxygenase (HO; n=5). Hemin significantly induced the activity of HO and Zn protoporphyrin IX (ZnPPIX) and significantly inhibited the activity of HO. Student’s t-test, ^**^P<0.01 for hemin group vs. control group; ^##^P<0.01 for ZnPPIX group vs. control group. ZnPPIX, Zn protoporphyrin IX; ACSF, artificial cerebrospinal fluid.

An icv hemin injection caused the HO activity to increase significantly. Injection of ZnPPIX icv significantly suppressed the activity of HO. Injection of ACSF icv had no effect on the activity of HO (Fig. 4).

### Effect of behavioral training on HO mRNA expression

**Figure 5:**
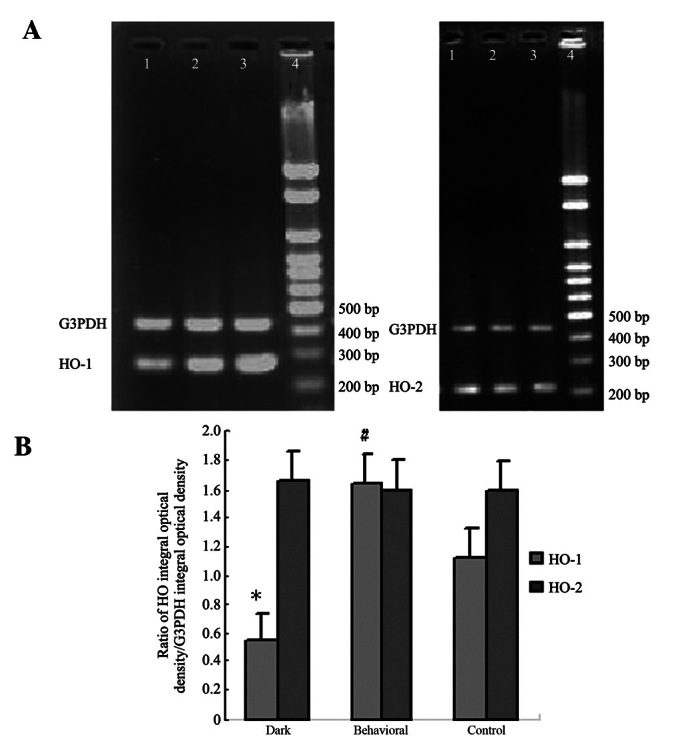
Effect of behavioral training on expression of heme oxygenase (HO) mRNA (n=5) with expression of HO mRNA detected by RT-PCR. (A) Lane 1, dark conditions group. Lane 2, control group. Lane 3, behavioral training group. Lane 4, DNA marker. (B) Behavioral training significantly upregulated and dark conditions significantly downregulated the expression of HO-1 mRNA. Student’s t-test, ^*^P<0.05 for dark conditions vs. control group, ^#^P<0.05 for behavioral training vs. control group.

Mice reared in dark conditions showed a low expression of HO-1 mRNA in hippocampal tissue. Behavioral training was found to significantly upregulate the expression of HO-1 compared to the control condition. There was no significant difference in HO-2 mRNA expression between the three groups (Fig. 5).

### Effect of behavioral training on HO protein expression

**Figure 6:**
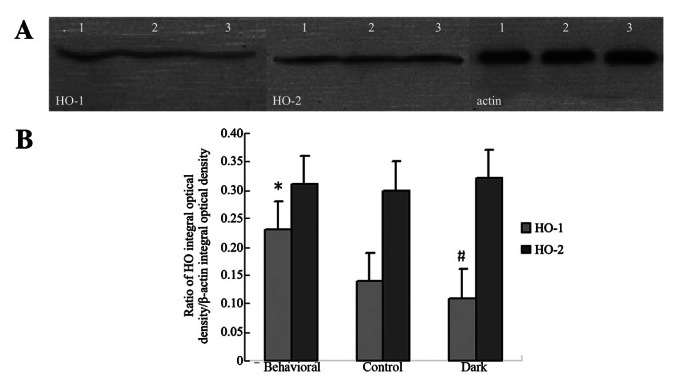
Effect of behavioral training on expression of heme oxygenase (HO protein, n=5). (A) Expression of HO detected by western blot analysis. Lane 1, behavioral training group. Lane 2, control group. Lane 3, dark conditions group. (B) Behavioral training significantly upregulated the expression of HO-1 protein and dark conditions significantly downregulated the expression of HO-1 protein. Student’s t-test, ^#^P <0.05 for dark conditions vs. control group; ^*^P<0.05 for behavioral training vs. control group.

The HO-1 protein expression of mice reared in dark conditions was lower than that of the control group. Behavioral training was found to significantly upregulate HO-1 protein expression. There was no difference in HO-2 protein expression between the groups (Fig. 6).

### Effect of behavioral training on activity of HO

**Figure 7:**
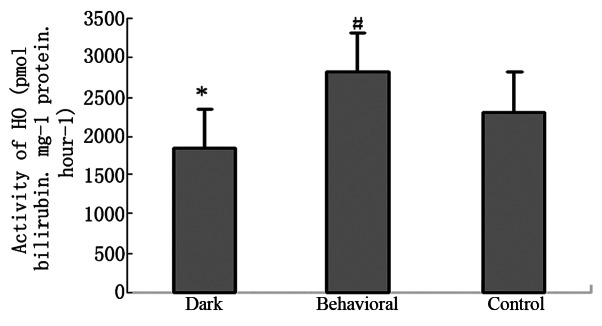
Effect of behavioral on activity of heme oxygenase (HO, n=5). Dark conditions significantly decreased the activity of HO and behavioral training significantly enhanced the activity of HO compared with the control group. Student’s t-test, ^*^P<0.05 for dark conditions vs. control group, ^#^P<0.05 for behavioral training vs. control group.

Compared with the control group, the HO activity of the dark-reared group was decreased. Behavioral training significantly increased its activity (Fig. 7).

## Discussion

HO-1 and 2 degrade heme to carbon monoxide (CO), biliverdin and ferrous iron. Endogenous CO is involved in long-term potentiation (LTP) and avoidance learning (12). A long-term activity-dependent presynaptic enhancement during LTP has also been found to be induced by CO (13). Biliverdin is believed to be the most powerful endogenous antioxidant, which efficiently scavenges peroxy radicals and protects cells from a 10,000-fold excess of hydrogen peroxide (14).

HO-2 is constitutively expressed in cells and previous research has shown that HO-2 has no effect on memory and learning ability (15). HO-1 is an inducible form of HO. It may be induced by many factors, including heme, heat shock, intensive light and ultraviolet exposure (7). The activity of HO may be blocked by metalloporphyrins, including chromium mesoporphyrin (CrMP), manganese protoporphyrin, manganese mesoporphyrin (MnPP), zinc protoporphyrin (ZnPP), tin mesoporphyrin and tin protoporphyrin (SnPP) (16). The expression of HO-1 protein and HO activity in the hemin icv injection group was significantly increased when compared with the control group. In the ZnPPIX injection group, the activity of HO decreased without any change in HO-1 protein expression. ACSF injected icv did not change the level of HO-1 protein or the activity of HO. This indicates that the cannula implanting operation itself had no effect on the expression of HO or its activity. Consistent with previous studies, none of the pretreatments affected the expression of HO-2 protein.

Behavioral tests were used to evaluate the effects on learning ability of altering HO-1 in mature mice. The data show that neither avoidance learning nor spatial navigation learning changed with changes in HO-1. Overall, in the present study, there was no correlation between learning ability and HO-1 in adult mice.

In order to determine the function of HO-1 in the immature mice, a dark-reared model was constructed. In this model, the mice were deprived of the stress of sound and light since they were young. It has been reported that stress affects multiple memory systems (17). There is some evidence to suggest that the intelligence quotient of children with impaired vision or hearing is significantly lower than that of children with normal vision (18,19). According to this theory, it was possible that the intelligence quotient of dark-reared mice was lower than that of control group, which were raised in normal conditions and the intelligence quotient of behavioral training mice was higher than that of control mice. Based on these models, we detected the expression of HO-1 and 2 by RT-PCR and western blot. Results showed that unlike HO-2, the expression of HO-1 in the dark-reared mice was lower than that of the control group. Moreover, the behavioral training group was found to significantly upregulate HO-1 expression, when compared with the control group. A similar change in HO activity was also observed.

The results of the present study suggest that the modification of HO-1 has no effect on established memory and learning abilities in adult mice whose brains have developed completely. On the other hand, the brain of a neonatal mouse is immature and its learning ability and memory are poor. It has been demonstrated that stimuli such as light and sound could promote the development of memory systems, which stimulates the expression of HO-1. HO-1 catalyzes heme to produce CO and biliverdin, which is involved in LTP and in protecting neurons from oxidative stress. In conclusion, the present findings suggest that the upregulation of HO-1 expression may facilitate the memory and learning ability of mice during development.

## Figures and Tables

**Table I:** Effect of intracerebroventricular (icv) injection with various treatments on the step-down test (n=10, means ± SEM); icv injection with various drugs had no effect on the results of behavior tests.

Group	Error number	Electric shock time (sec)	Step-down latency (sec)	Time remaining on platform (sec)
ZnPPIX	1.8±0.41	30.9±15.50	71.9±30.17	158.2±30.10
Hemin	2.5±0.38	22.4±5.31	72.3±30.26	148.1±29.82
ACSF	1.9±0.28	44.0±22.42	83.6±31.05	120.5±34.12
Control	2.4±0.51	15.6±5.09	86.7±28.84	168.7±32.86

ZnPPIX, Zn protoporphyrin IX; ACSF, artificial cerebrospinal fluid.

**Table II:** Effect of intracerebroventricular (icv) injection with various treatments on swim speed (n=10, means ± SEM).

Group	Swim speed (cm/sec)
ZnPPIX	22.51±4.08
Hemin	26.34±3.37
ACSF	23.78±3.56
Control	24.04±5.36

ZnPPIX, Zn protoporphyrin IX; ACSF, artificial cerebrospinal fluid.
